# Full scale tests of various buried flexible structures under failure load

**DOI:** 10.1038/s41598-022-04969-7

**Published:** 2022-01-25

**Authors:** Adam Wysokowski

**Affiliations:** Road, Bridge and Railway Department, Faculty of Civil Engineering, Architecture and Environmental Engineering, University of Zielona Góra, Prof. Z Szafrana St. No. 1, Building A-8, 65-417 Zielona Gora, Poland

**Keywords:** Civil engineering, Mechanical properties

## Abstract

The aim of the tests described in the paper was conducting a displacement and stress analysis of four buried flexible structures made in different technologies under failure load. Generally, the laboratory tests carried out on in full scale confirms that all four culvert models of the work safely despite the reduction of the backfill layer over the structures to 0.3 m (less than according to standard recommendations) and increasing the load to 1960 -2000 kN (almost 4 times more than according to standards and recommendations). In the case of a PE plastic pipe, the main measured parameter was displacement and for other steel research models it was the stress. The obtained maximum values of these parameters were compared with the permissible standard values. Maximum displacement values were recorded in the crowns of the structures up to 12.57 mm for PE plastic pipe model. The maximum stresses for steel structures were 85.28 MPa (for corrugated steel pipe), 111.5 MPa (Box Culvert) and 447.9 MPa (Multi Plate non-circular structure). Despite the exceed stress in the case of Multi Plate structure, the steel structure did not lose its stability. The analyses carried out were aimed at determining database, which in its assumption, allows the verification of calculations performed by a numerical method such as FEM.

## Introduction

The article attempts to evaluate the comparison values of the culvert displacements and stresses made in four different technologies with the permissible standard values based on laboratory tests on a natural scale. Models were made using PE plastic and corrugated steel sheets. In this case, the results of the structural tests were compared under a failure load.

**Figure 1:**
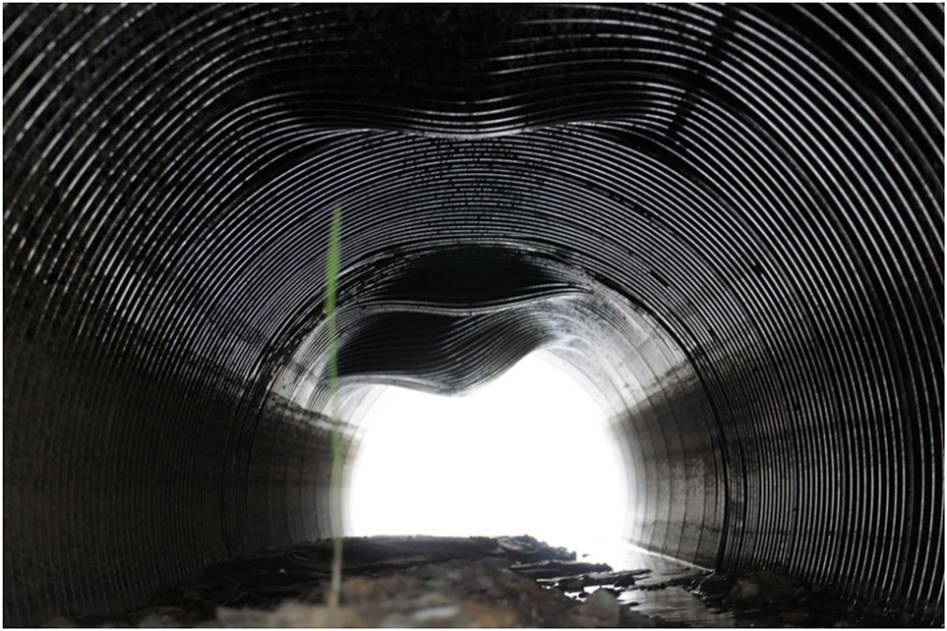
An example of damage caused by overloading a road buried flexible steel structure. Visible deformations in the places of the vehicle axis run-up.

An example of damage caused by an overload of buried flexible structure is shown in Fig. 1.

The topic described in the paper is very important due to the fact that in recent years, research and analysis of culverts under various types of loads are still being carried out. This is related to both culverts in situ tests^1–4^, as well as numerical analysis of different load cases^5–9^.

In the case of buried flexible structures, the impact of the backfill on the soil-shell interaction is less pronounced than in the case of rigid structures. This is because in these structures, once the ground is made, a natural vault is formed in the backfill, limited from the top by the road surface and from the bottom by the curvature of the structure. This phenomenon is called "vaulting", although in the natural situation it occurs as a result of creating a hole inside the stabilized natural soil and not in the embankment of the soil-covered structure under construction^10^. In such a situation, the dead load of the layers (soil backfill and roadway substructure) and the live loads cause much less reaction on the foundation than in a rigid (classic) vaulted structure. In the case of buried flexible structures, the interaction of the flexible structure with the soil backfill is employed. Therefore the main load-bearing structure is the soil backfill as well as the reinforcing geotextiles. In the case of rigid culverts, the backfill is only the filling without significant interaction.

Despite the extensive theoretical research that has been carried out to model soil-structure interactions leading to many mathematical relationships and empirical equations, most of them present a shortcoming in considering the actual soil-shell-interaction response. One common way to obtain real information about this interaction is to develop a physical model capable of providing various conditions^11,12^. Such model allows the measurement of most parameters related to the behavior of the buried flexible structure with high accuracy. At the same time, the model allows, to measuring parameters under various service loads (static, dynamic and fatigue), and also to determine the mechanism of failure of such structures under an extreme live load. It is the most accurate method of performing such analyzes^13^, which can certainly be supplemented with the most modern FEM analyzes, including heterogeneous models of structure material^14^.

The article presents the results of analyzes of four full-size laboratory cases made in different technologies. In all cases, the applied loads exceeded the assumed standard loads by 400%. The studies provide a good basis for assessing the accuracy and reliability of the commonly used finite element analysis methods^15,16^.

It should be noted that compared to numerical analysis, building, testing, and then the demolition of the natural scale model requires a very large financial outlays. For this reason, such studies are conducted less frequently in the world.

## Materials and methods

Four research models made in different material technology were tested on a natural scale. The main purpose of the research was to compare the displacement and stress values of structures under failure load. Tests were carried out on the station for static, dynamic and fatigue tests, which consists of a reinforced concrete foundation with a length of 80.0 m and a width of 20.0 m together with a hall and a steel frame constituting a retaining construction for hydraulic load-inducing devices.

Research models were constructed in all technological steps of the buried structure based on the applicable transport engineering standards and regulations, included additionally geometric control of the test models, mounting sensors and gauges on the structures and soil backfilling with mechanical compaction with 30.0 cm thick layers. Displacement and stress measurements were carried out using sensors and induction gauges in characteristic points of the structures. The applied loads were in accordance with the Polish transport engineering standards. The load variant replacing the railway load was used in the laboratory tests. Failure tests of culvert models were carried out for several different values of load forced by hydraulic actuators and for different values of backfill layer over the structures. The article describes the results for maximum loads and backfill layer equal 0.3 m. The backfilling material was well-graded soil with a maximum grain size of 32 mm. The basic parameters of soil backfill are summarized in Table 1.

**Table 1:** Properies of soil backfill.

Parameter	Unit	Value
Sand indicator	%	92.5
Optimal moisture content	%	9.5
Compaction by proctor	g/cm^3^	1.990
Bulk density	g/cm^3^	1.74
Bearing capacity CBR (5,0)	%	17.0
Aggregate abrasion by the Los Angeles method	%	26.0

The aim of laboratory tests on a natural scale was to create a knowledge base on the behavior of buried structures under various types of loads. This database, in its assumption, allows the verification of calculations performed by a numerical method such as FEM.

### Description of PE plastic and steel corrugated models

**Figure 2:**
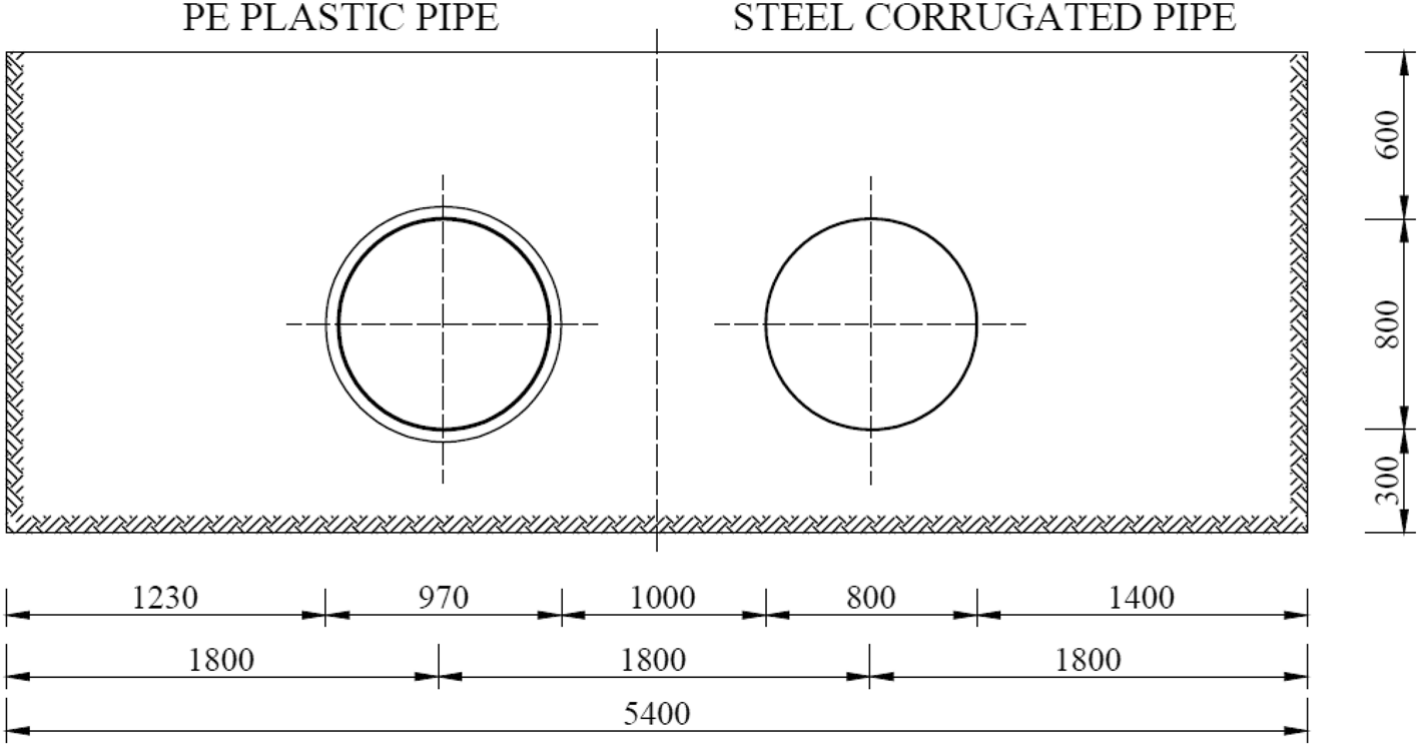
Cross-section of the research models: PE plastic pipe and steel corrugated pipe.

The research covered two culverts with a diameter of 0.80 m and a length of 13.70 m. The first research model was made of a PE plastic pipe. The second model was a flexible corrugated steel pipe. The soil conditions for both models were similar due to constant monitoring of the degree of compaction of soil backfill layers and of the lateral zone of the structures. Figure 2 shows a cross-section of the research models.

**Figure 3:**
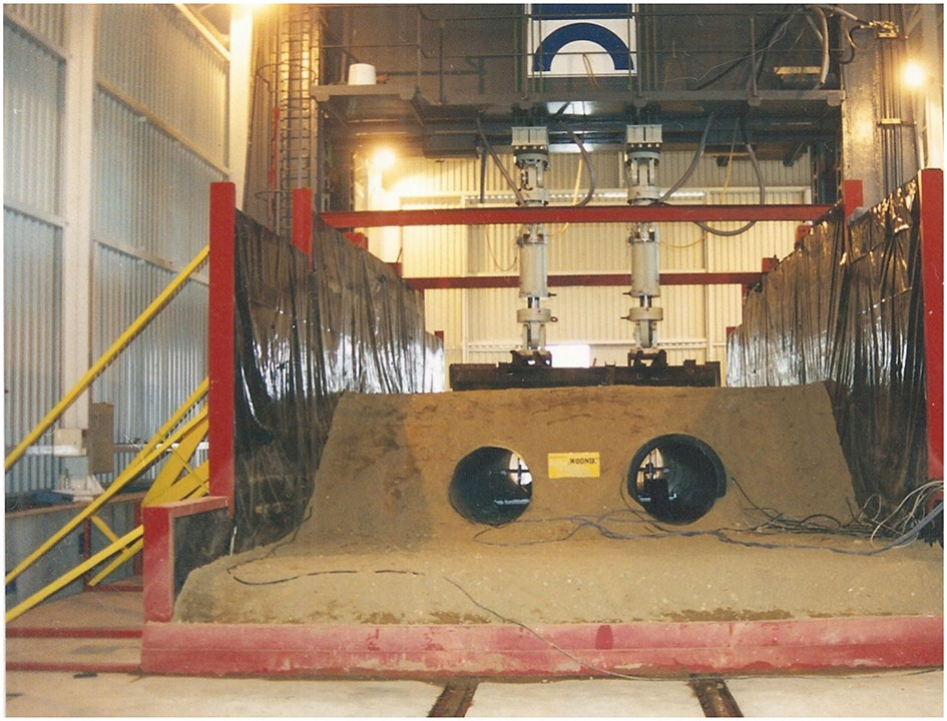
General view of the research models after soil backfill.

Figure 3 present general view of test model for research.

The basic parameters of PE plastic pipe are summarized in Table 2. Table 3 contains the list of the basic parameters of pipe made of corrugated steel sheets.

**Table 2:** Parameters of the pipe made of PE plastic.

Parameter	Unit	Value
Nominal diameter	(mm)	800.0
Outside diameter	(mm)	970 $\pm$ 2%
Inside diameter	(mm)	800 $\pm$ 2%
Area	(m^2^)	0.50
Period of corrugation	(mm)	140.0
Ring stiffness	kPa	6.0
Density	(g/cm^3^)	0.942

**Table 3:** Parameters of the pipe made of corrugated steel sheets.

Parameter	Unit	Value
Nominal diameter	(mm)	800.0
Sheet thickness	(mm)	2.0
Area	(m^2^)	0.50
Weight	(kg/m)	47.8
Tensile strength	(MPa)	270.0
Yield point	MPa	250.0

The laboratory failure tests were carried out for the following maximum loads:

- maximum pressure force: F_max_ = 1960 kN.
- maximum force per actuator:F_max_ / 2 = 980 kN.

**Figure 4:**
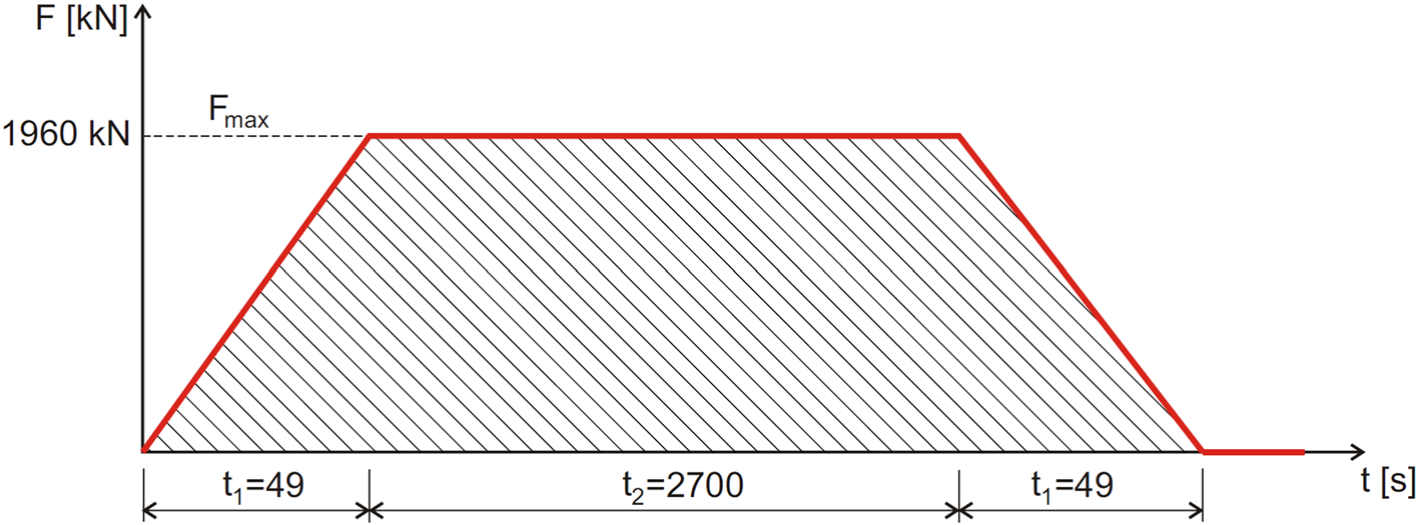
Diagram of the failure load.

Failure tests of research models were carried out according to the diagram shown in Fig. 4.

**Figure 5:**
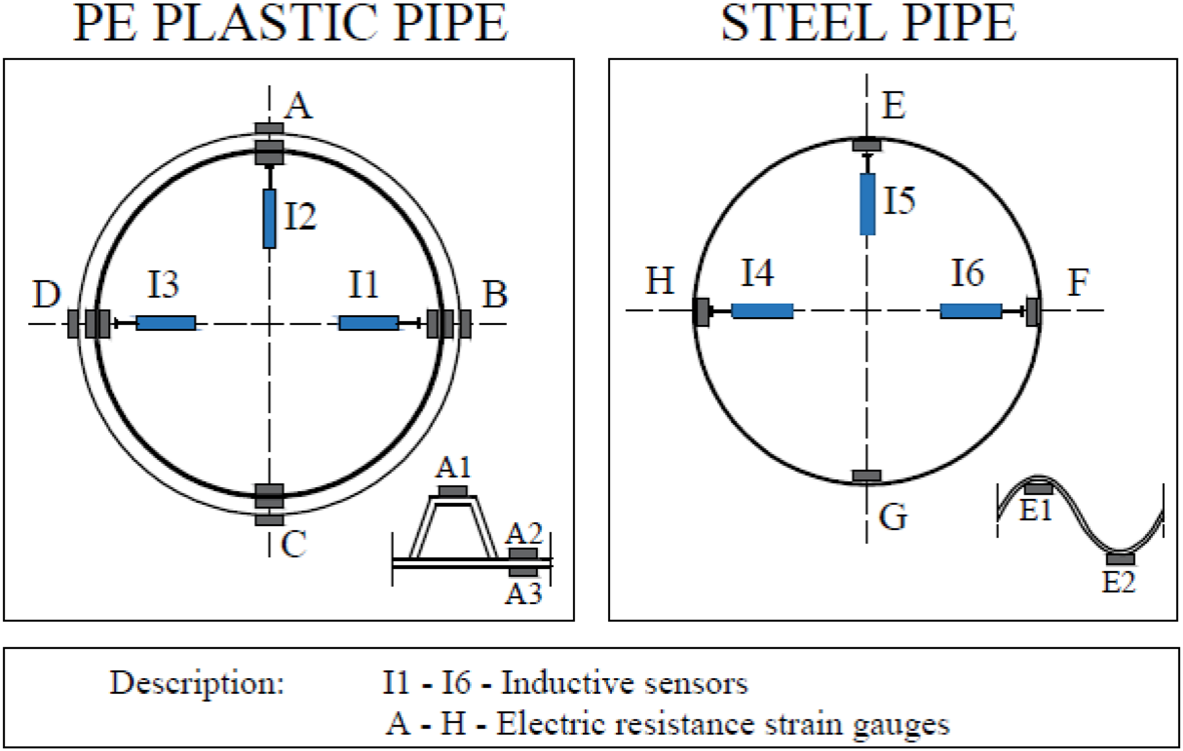
Arrangement of induction sensors and strain gauges for the testing models.

Displacement measurements were carried out using induction gauges located in the vertical axis (in the crown) and in the horizontal axis (on both opposite side walls). Stress measurements were carried out using electric resistance strain gauges located in the vertical axis and in the horizontal axis (on both opposite side walls). The arrangement of the induction sensors and strain gauges is shown in Fig. 5.

### Box Culvert test model

**Figure 6:**
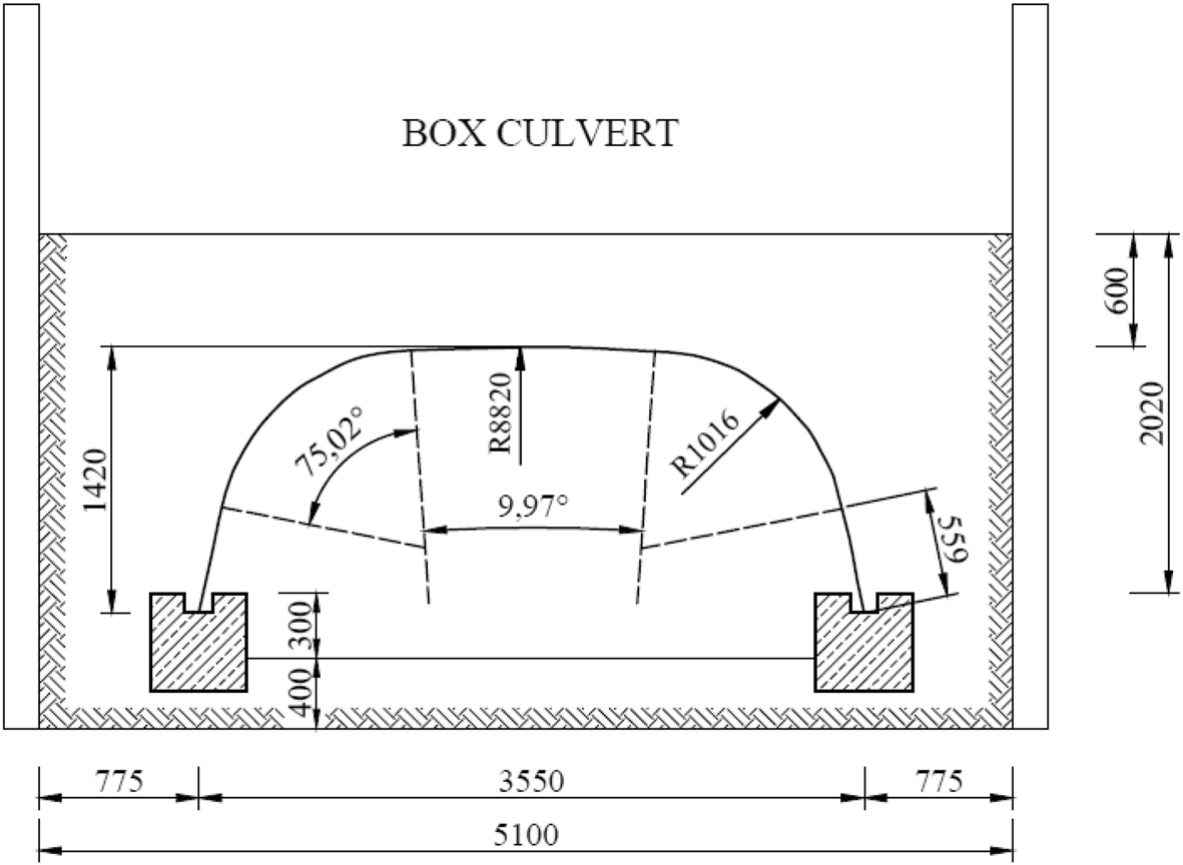
Cross-section of the research model: Box Culvert.

The next stage of research concerned Box Culvert model under failure load. The research covered a culvert with a span of 3.55 m and a height of 1.42 m. The steel structure was additionally reinforced by special ribs made of steel plates located on the top section of perimeter—in the crown. Figure 6 shows a cross-section of the research model.

**Figure 7:**
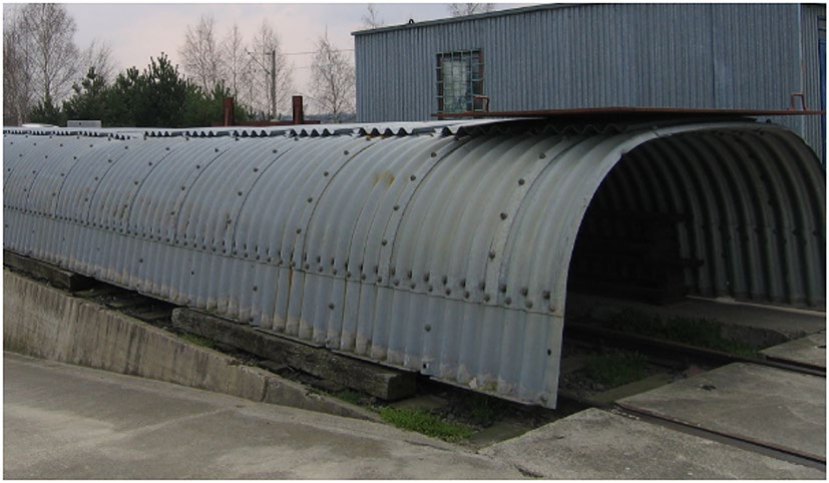
General view of the tested steel box structure.

**Figure 8:**
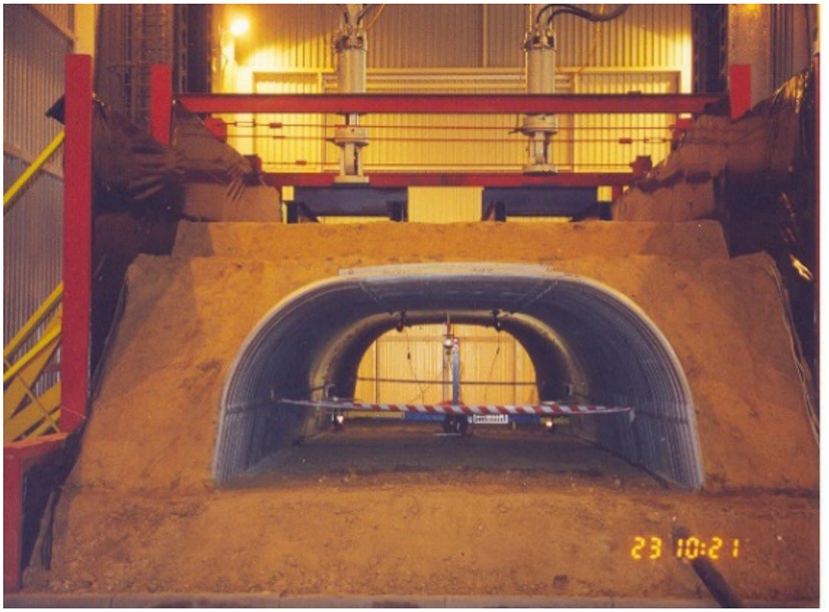
General view of the research model after soil backfill.

A general view of the steel structure for testing is shown in Fig. 7. Figure 8 presents a general view of the research model after soil backfill.

Type of the model profile is a box with open cross-section. Other basic parameters of the tested structure are summarized in Table 4.

**Table 4:** Parameters of the Box Culvert research model.

Parameter	Unit	Value
Thickness of the steel plate	(mm)	5.0
Corrugation of the steel plate	(mm)	150.0 × 50.0
Length	(m)	13.7
Tensile strength	(MPa)	270.0
Yield point	(MPa)	250.0
Cross-section of concrete foundations	(m)	0.50 × 0.50

The laboratory failure tests were carried out for the following maximum loads:

- maximum pressure force: F_max_ = 1990 kN,
- maximum force per actuator: F_max_/2 = 995 kN.

The speed of the load was 40 kN/s with the time of the maximum load T = 600 s.

The data collecting equipment consisted of 22 electric resistance strain gauges in 11 locations, with a strain gauge on the top and bottom of the corrugation and 3 inductive sensors used for vertical and horizontal displacement measurements.

**Figure 9:**
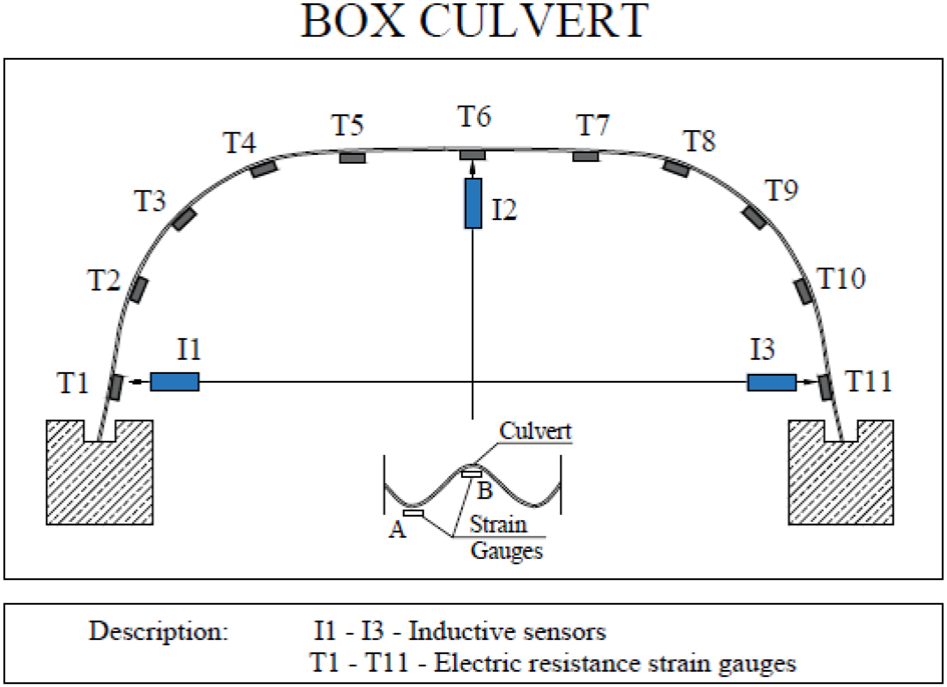
Arrangement of induction sensors and strain gauges for the Box Culvert model^1^.

Figure 9 presents a schematic layout of strain gauges and induction sensors around the corrugated Box Culvert model^1^.

### Description of the multi plate model

**Figure 10:**
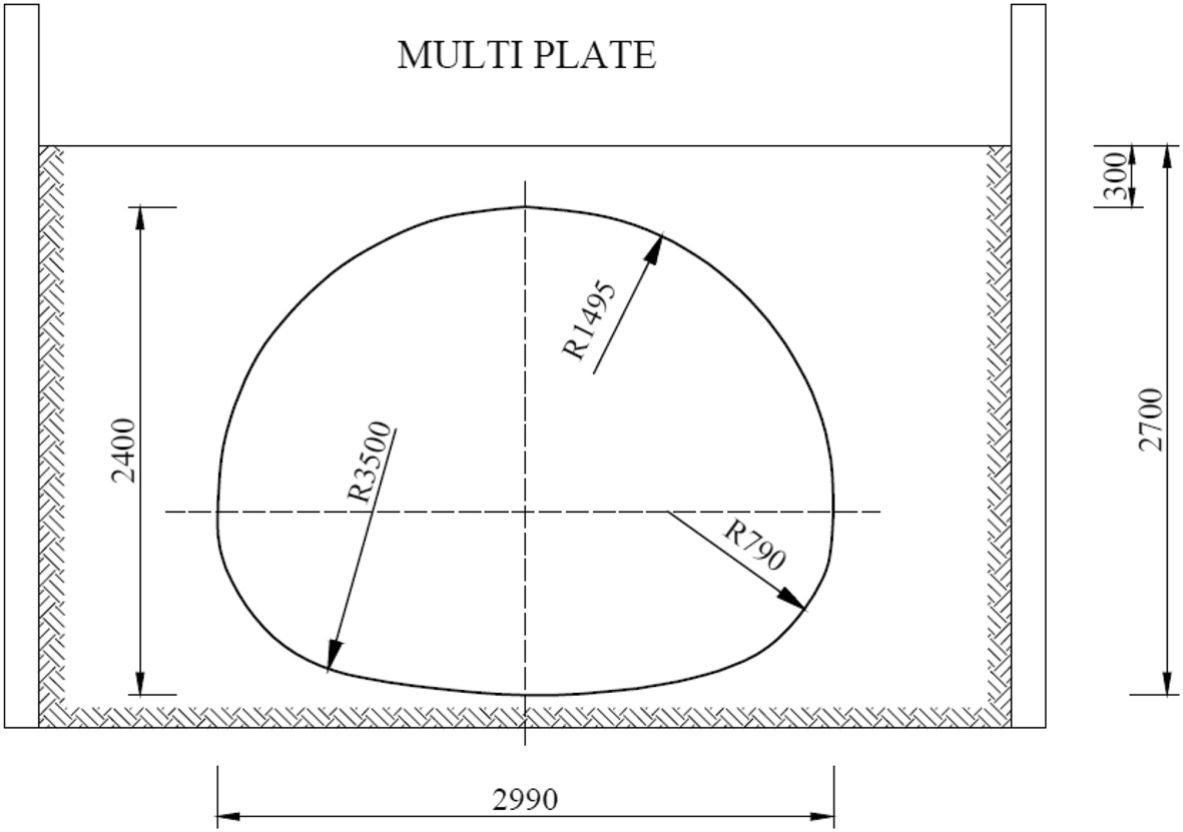
Cross-section of the research model: multi plate.

Another research model in natural scale was Multi Plate structure made of corrugated steel sheets. The research covered a culvert with a span of 2.99 m and a height of 2.40 m. Other parameters of the research model are shown in Table 5. Figure 10 shows a cross-section of the Multi Plate model.

**Table 5:** Parameters of the multi plate research model.

Parameter	Unit	Value
Thickness of the steel plate	(mm)	3.75
Width of the steel plate	(mm)	990.0
Corrugation of the steel plate	(mm)	150.0 × 50.0
Length	(m)	14.4
Tensile strength	(MPa)	270.0
Yield point	(MPa)	250.0

**Figure 11:**
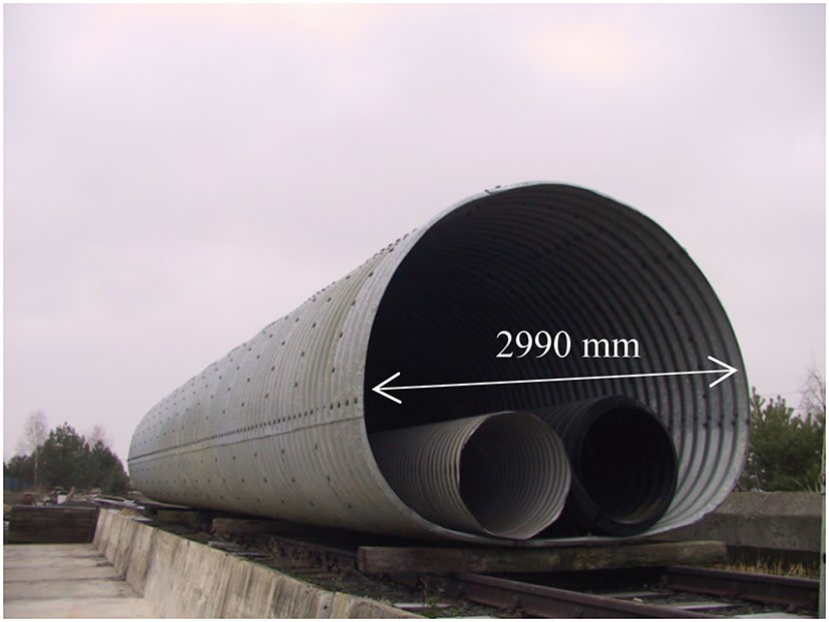
General view of the tested steel Multi Plate structure including the two pipes from the chapter 2.1.

**Figure 12:**
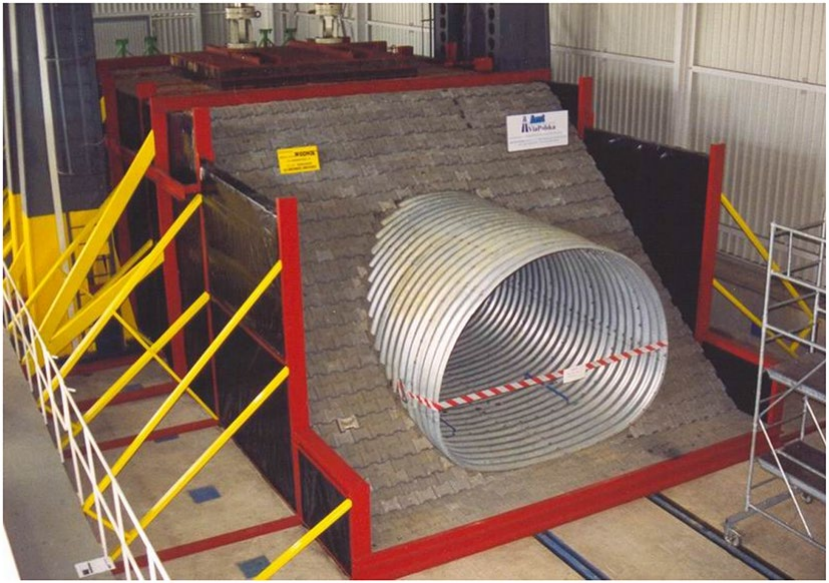
General view of the complete research model.

General view of the steel Multi Plate structure for testing is shown in Fig. 11. Figure 12 presents a general view of the research model ready for testing.

Described laboratory failure tests were carried out for the backfill layer over the structure equal 0.3 m and for the following maximum loads:

- maximum pressure force: F_max_ = 2000 kN,
- maximum force per actuator:F_max_ / 2 = 1000 kN.

**Figure 13:**
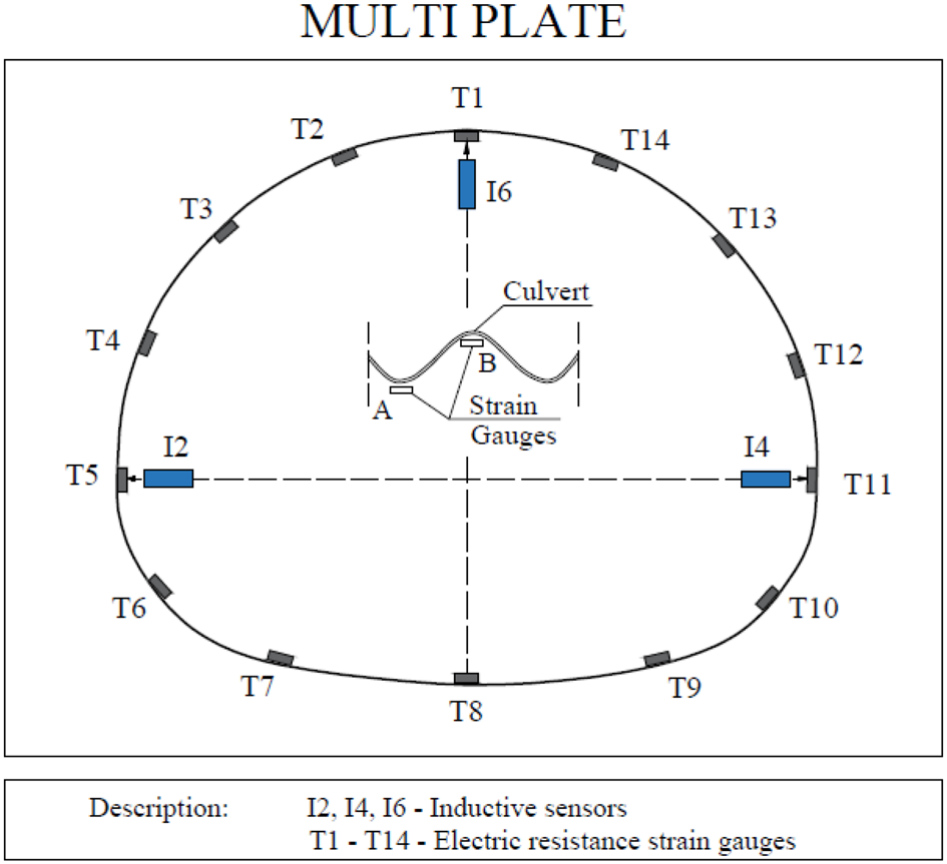
Arrangement of induction sensors and strain gauges for the multi plate model.

Displacement measurements were carried out using three inductive sensors located in the vertical axis (in the crown) and in the horizontal axis (on both opposite side walls). Stress measurements were carried out using 28 electric resistance strain gauges in 14 locations, with a strain gauge on the top and bottom of the corrugation. The strain gauges were located on the perimeter of the research model at equal distances of 631 mm. The arrangement of the induction sensors and strain gauges is shown in Fig. 13.

### Design standards

According to the Polish Standards in the case of buried structures made of PE plastic, an important parameter is the allowable deflection, which is equal to 3%. When designing this type of structure, it is necessary to estimate the value of the maximum deflection during use of the object and compare it with the permissible value. Deflection of the structure results from the displacement and the span ratio^17^.

During the implementation of culverts made of corrugated steel sheets, compliance with the permissible values of wall stresses must be checked. The allowable stress value in this case is equal to the tensile strength of the steel from which the buried structure was made.

### Ethical approval

This article does not contain any studies with human participants or animals performed by any of the authors.

## Results and analysis

### Test results

**Figure 14:**
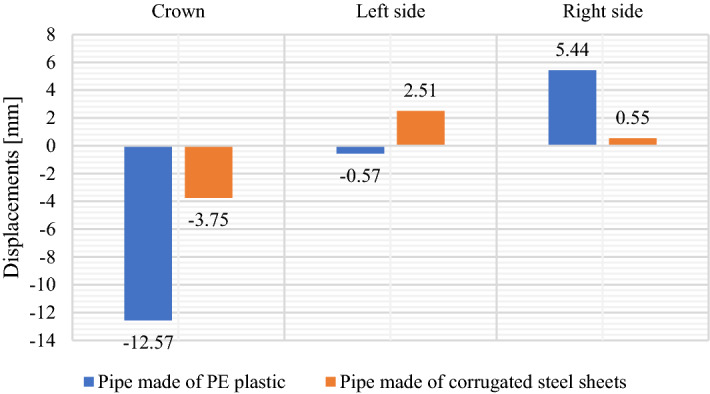
Maximum displacement values of the research models: PE plastic pipe and corrugated steel pipe.

The results of displacements and stress measurements concern characteristic points of the structures. Test results were graphically developed for all research models under failure load. Maximum measured values in individual sensors are shown in Fig. 14.

Figure 8 shows displacement diagram recorded for structures made of PE plastic pipe and corrugated steel pipe. Presented displacements results pertain to inductive sensors located in the crown of the structures and in the horizontal axis on both opposite side walls (Fig. 5) marked as I1, I2 and I3 for PE plastic pipe and as I4, I5 and I6 for pipe made of corrugated steel sheets.

**Figure 15:**
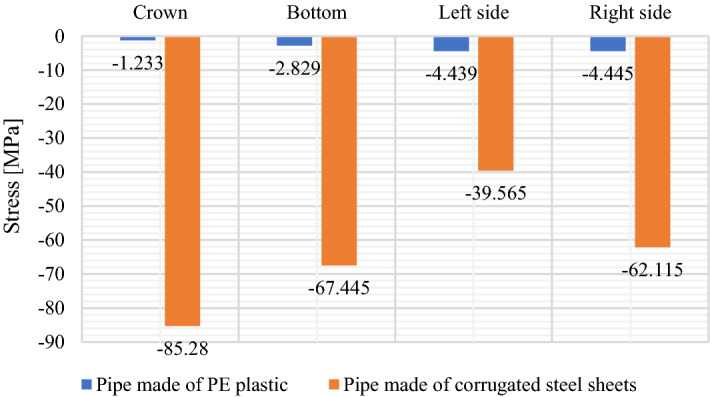
Maximum stress values in the research models: PE plastic pipe and corrugated steel pipe.

Figure 15 shows stress diagrams recorded for the strain gauges located in the horizontal axis, crown and bottom of the structures (Fig. 4).

**Figure 16:**
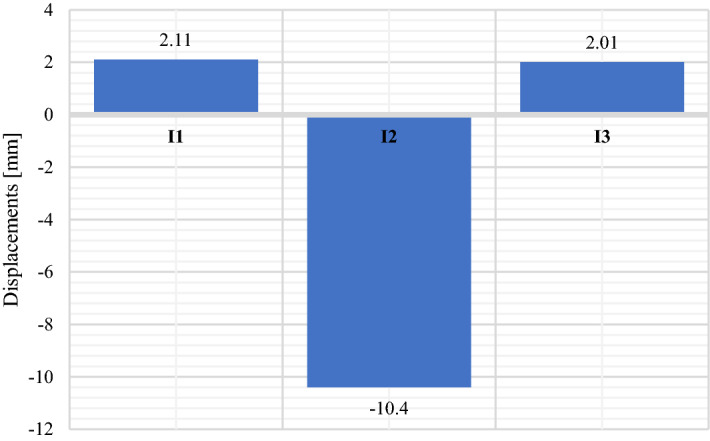
Maximum displacement values of the research model: Box Culvert.

Figure 16 shows maximum displacement values recorded for the induction sensors (I1 – I3) located in the horizontal axis and crown of the Box Culvert model (Fig. 9).

**Figure 17:**
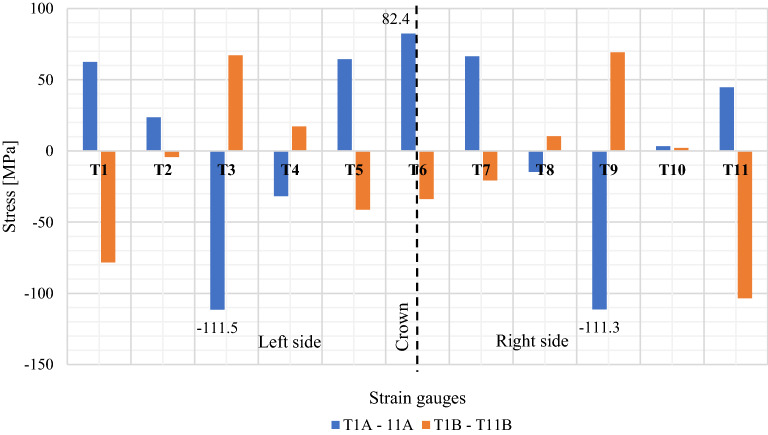
Maximum stress values in the research model: Box Culvert.

Figure 17 shows stress diagram recorded for Box Culvert model. Presented maximum stress results pertain to strain gauges marked as T1A-T11A and T1B-T11B (Fig. 6).

During the study also made an attempt to “destroy” the construction of box culvert. Due to the limited maximum vertical load (with a maximum load of 2000 kN the structure showed no damage), the top layer of the soil backfill was removed for the destruction test, and the load was applied directly to the upper surface of the steel structure with only 10.0 cm soil cover.

**Figure 18:**
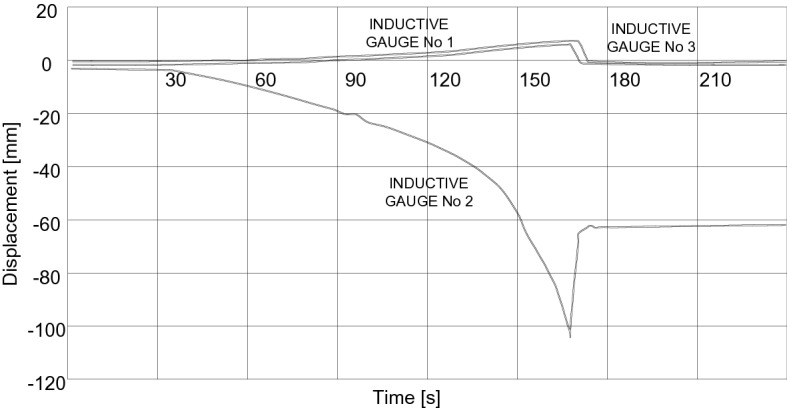
Example of vertical displacement under destructive load for Box Culvert structure.

In Fig. 18 are shown vertical displacements under destructive load for Box Culvert structure (for three inductive gauges).

**Figure 19:**
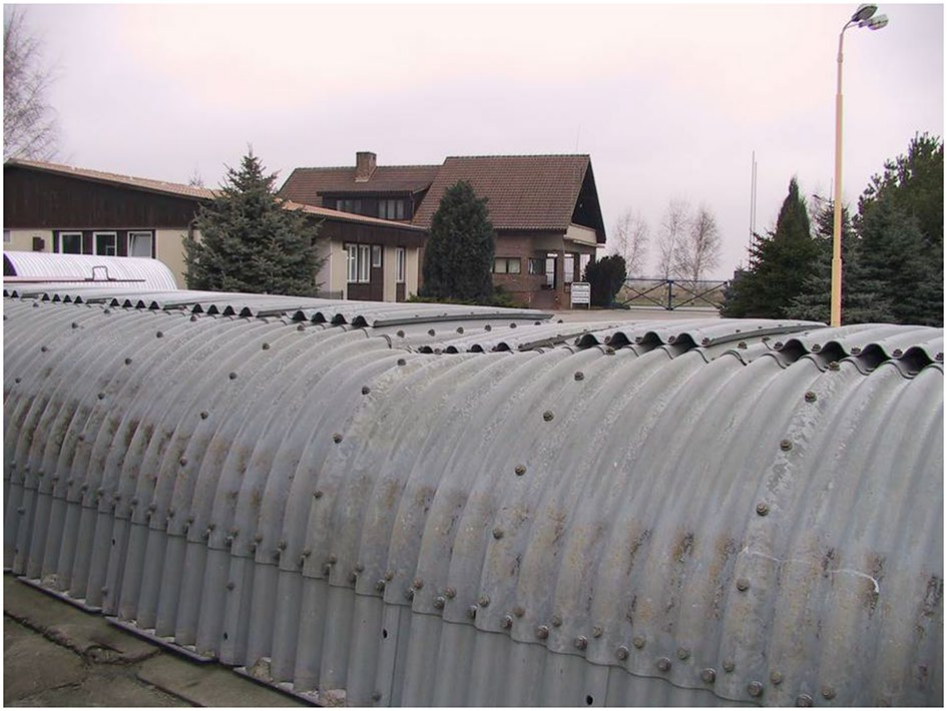
Local damage where the stiffness of the structure changes after the tests.

**Figure 20:**
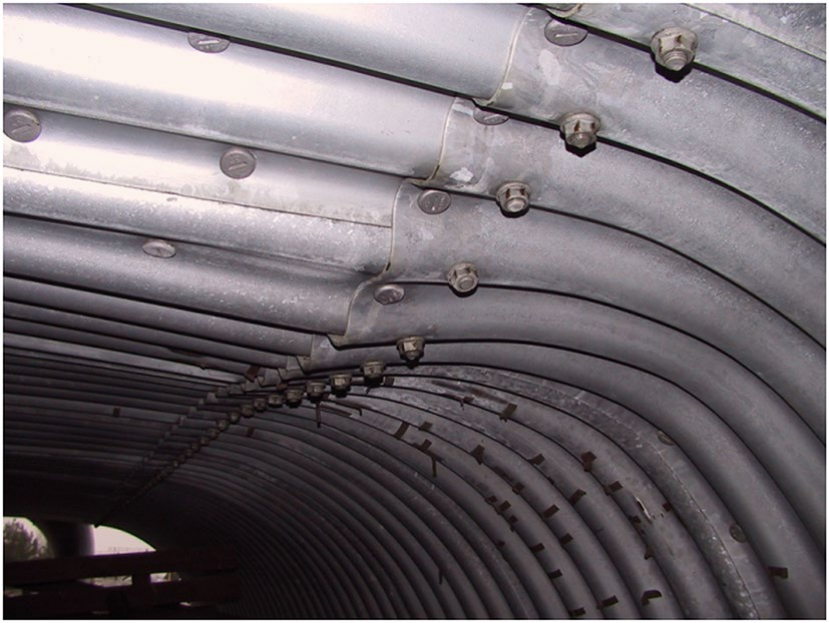
View of damage caused by concentrated load applied directly to the steel structure.

Figures 19 and 20 show the effect of an attempt to destroy the box culvert structure by applying a load directly to the crown of steel structure (with only 10.0 cm soil contact layer).

**Figure 21:**
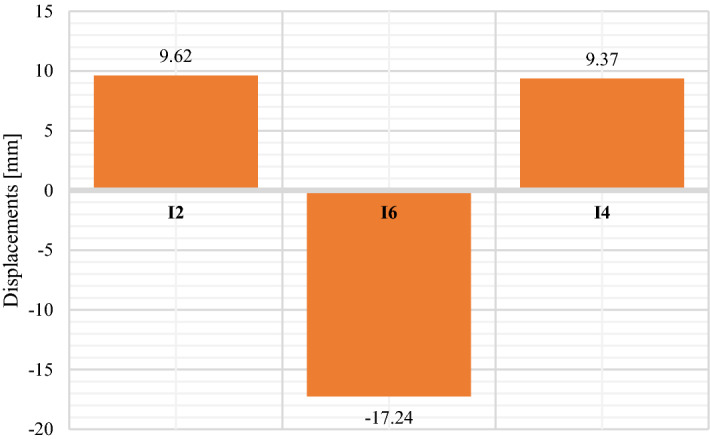
Maximum displacement values of the research model: multi plate.

Figure 21 presents displacements results pertain to inductive sensors located in the crown of the Multi Plate structure and in the horizontal axis on both opposite side walls marked as I2, I6 and I4 (Fig. 13).

**Figure 22:**
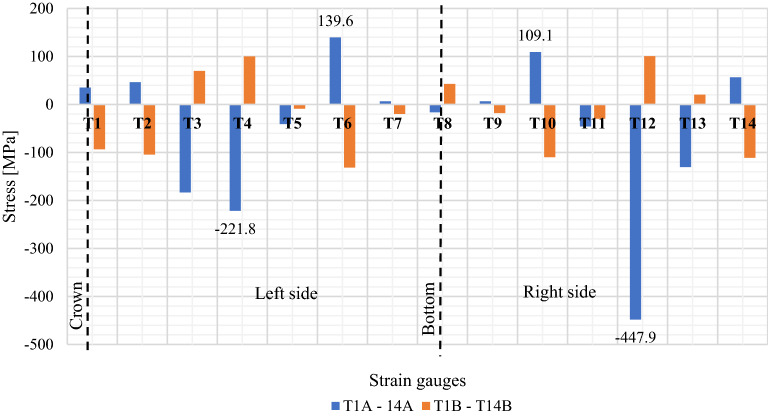
Maximum stress values in the research model: multi plate.

Figure 22 shows stress diagram recorded for Multi Plate model. Presented maximum stress results pertain to strain gauges marked as T1A-T14A and T1B-T14B (Fig. 13).

### Analysis of test results

The laboratory tests indicate the correct operation of all tested models. During the research, no factors were observed that would affect the need to change the test program and results. The obtained test results were subjected to appropriate analysis. For its ease, additional table was prepared with maximum results. Table 6 presents a summary of the maximum recorded values of the stress and displacements of the structures.

**Table 6:** Maximum stress and displacement values of the tested models.

Parameter	Research model							
	PE plastic pipe		Corrugated steel pipe		Box Culvert		Multi plate	
	Value	Sensor	Value	Sensor	Value	Sensor	Value	Sensor
Displacement (mm)	−12.57	I2	−3.75	I5	−10.4	I2	−17.24	I6
Stress (MPa)	−4.45	B3	−85.28	E2	−111.5	T3	**−447.9**	T12

Significant values are in bold.

Table 7 compares the maximum displacement and stress values measured for the research culvert models with the permissible standard values.

**Table 7:** Comparison of maximum displacement and stress values with the permissible standard values.

Research model	Parameter	Value	Permissible standard value
PE plastic pipe	Deflection	(12.57:800.0) × 100% = 1.57%	3%
Corrugated steel pipe	Stress	85.28 MPa	270 MPa
Box Culvert	Stress	111.5 MPa	270 MPa
Multi Plate	Stress	**447.9 MPa**	270 MPa

Significant values are in bold.

Laboratory tests of culverts in natural scale under failure load with backfill layer over the structures equal 0.3 m, did not show irregularities in the sense of stability and safety of work of the research models. As a result of the analysis of the displacement and stress values, the permissible standard values of these parameters were not exceeded, except for the stress value measured by the T12 sensor for the MultiPlate model (Fig. 13). Extreme displacement and stress values occur at those measuring points where the maximum values for other load variants are recorded^4,18^. Similar course of internal forces is proof that the tested structures do not show a tendency to excessive deformation and do not change its rigidity during the load process.

Lowering the backfill layer below the minimum recommended value and increasing the load value imposed by the actuators did not cause complete destruction or deformation that would prevent the use of the structures.

## Conclusions

In the article, an analysis of the behavior of buried structures made in different technologies under failure load was carried out. The conducted analysis showed that all tested structures behave in a safe manner. Almost symmetrical layout of measured stresses and displacements, it proves that the maximum load possible to obtain from actuators of 1960–2000 kN does not affect the change in stiffness and also the symmetry of the tested structures.

The results concerning the multiplate design were surprising. Despite exceeding the allowable stresses in the structure key (449.7 MPa), the thin-walled steel structure did not lose its stability. Probably the stability of structure results from the phenomenon of "soil interaction" with the thin-walled steel structure. This phenomenon is based on the redistribution of loads in the model under the influence of increasing load. Thus, the stresses in the soil on both sides of the shell increase through lateral deformation of the steel structure, which has an influence on the stresses / displacements in the crown of the structure. Additionally, no cracks were observed on the surface of the steel structure.

The comparison of the maximum displacement and stress values with the permissible standard values confirms the considerable load capacity of buried structures. In the case of accurate execution and compaction of backfill, the structures work correctly and safely even for a reduced backfill value over the structure to 0.3 m and at increased loads. The load value from 1960 to 2000 kN significantly exceeds the maximum load resulting from Polish bridge design standards equal to 500 kN (50 tons) and the maximum load according to NATO Standards (STANAG 2021 Military Load Classification of Bridges, Ferries, Rafts and Vehicles) equal to 600 kN (60 tons).

A damage of flexible box structure was only possible under direct load placed on the steel structure with 10.0 cm soil cover (contact layer). It reflects that soil-structure interaction is a key for performance of these type of structures or pipes.

In the case of such hybrid structures, the susceptibility of the cover means that the backfill of the soil is clearly dominant as a structural substructure. An internal vault is created in the ground backfill which transfers the operational loads from the ground upwards away from the coating—what is referred as the “vaulting effect”. This occurs mainly in the case of thin curved flexible structures—which naturally reflect the force flow in the soil. In the case of box-shaped objects, this phenomenon is limited, which is clearly visible in the analysis of the results. In addition, the vaulting effect occurs as the thickness of the upper layer increases.

In the initial phase, the flexible structure is merely only a formwork for creating the backfill geometry.

The behavior of soil-structure interface can be crucial to the overall response of a soil-structure system. The numerical simulation of soil-structure interaction problem requires proper modeling of the interface^19^.

Measurement data obtained during the laboratory tests in natural scale, in addition to the basis for scientific studies, give the possibility of more accurate calibration and verification of calculations performed by analytical and numerical method such as FEM. The analysis confirms that buried flexible steel structures are an alternative solution for traditional engineering facilities^20^.
